# The effect of evidence-based discharge planning on the health outcomes of stroke patients with dysphagia: a prospective cohort study

**DOI:** 10.3389/fneur.2025.1707847

**Published:** 2026-01-07

**Authors:** Yanping Lei, Min Li, Zhenxing Liu, Yan Zhang, Rong Huang, Dandan Chen, Xiaoling Liu, Xiao Fu, GangRong Huang, Jun Wei

**Affiliations:** 1 The First College of Clinical Medical Science, China Three Gorges University, Yichang, China; 2 Yichang Central People's Hospital, Yichang, China; 3 Hubei Province Clinical Medical Research Center for Rare Diseases of Nervous System, Yichang, China; 4 Yiling People's Hospital, Yichang, China

**Keywords:** stroke, dysphagia, discharge planning, unplanned readmission, aspiration, self-management, safe feeding

## Abstract

**Aim(s):**

To evaluate the effects of an evidence-based discharge planning on the health outcomes of stroke patients with dysphagia.

**Design:**

Prospective cohort study.

**Methods:**

This study was conducted with stroke patients who were diagnosed with dysphagia and scheduled to be discharged to their homes. A total of 90 eligible patients were consecutively enrolled to the exposed group and non-exposed group. The exposed group received an evidence-based discharge planning with follow-up at 7 and 30 days after discharge. The non-exposed group received routine care with the same length of care and follow-up as the exposed group. Trained research assistant collected all patients’ baseline data on admission (T0), recorded unplanned re-admission and aspiration, evaluated the discharge readiness, self-management ability and safe feeding on the day of discharge (T1), 7 days post-discharge (T2), and 30 days post-discharge (T3).

**Results:**

Compared with the non-exposed group, the exposed group showed significantly lower rate of unplanned readmission, lower incidence of aspiration, improvement in discharge readiness, self-management ability, and safe feeding scores.

**Conclusion:**

An evidence-based discharge planning can significantly reduce unplanned readmission rate and aspiration in stroke patients with dysphagia, and significantly improving patients’ discharge readiness, self-management ability and safe feeding. It provides clinical care with a clear and effective guideline plan for the management of patients with dysphagia in stroke.

**Clinical trial registration:**

The study has been registered with the Chinese Clinical Trial Registry (http://www.chictr.org.cn) under registration number ChiCTR24000087983.

## 1 Introduction

Stroke is the first cause of death and leading cause of adult disability in China (1). Approximately 17.04 million individuals aged 40 years and older in China had experienced or are currently affected by stroke, with about 1.94 millions of them died as a result (2) Approximately 2 million new cases of stroke are reported annually in China (3). Dysphagia is a common and serious complication after stroke (4), which occurs in about 64–78% of acute stroke patients, and may also develop in 40–81% of recovering patients (5). Dysphagia often leads to aspiration, malnutrition, increased risk of infection, prolonged hospitalization, and unplanned re-admissions. Within 6 months of onset, stroke patients with dysphagia have a 23% higher mortality rate and poorer quality of life than those without dysphagia (6).

Stroke patients with dysphagia and their families face serious challenges during the patient’s transition from hospital discharge to home, such as lack of self-care knowledge, poor treatment adherence and insufficient nursing resources. A cohort study of approximately 2 million stroke patients in the United States over a 6-month period showed that 12.4% of ischemic stroke patients were readmitted within 30 days after discharge, of which approximately 90% were unplanned readmission (7). In China, the 30-day readmission rate of stroke patients is higher than that in developed countries. The research involving 50,912 stroke patients showed that the 30-day unplanned readmission rate of stroke patients reached 28.8%. Recent studies also showed that the 30-day readmission rate of acute ischemic stroke patients is about 30% (8). This leads to deterioration of the patient’s condition and increases the risk of unplanned readmission, which seriously affects patient safety (9, 10). Therefore, it’s critical to help patients safely transition from hospital to home by standardizing the implementation of discharge planning for stroke patients with dysphagia.

Discharge planning aim to help patients receive continuous and complete care through the cooperation of a multidisciplinary team provided by the hospital and the patient’s family (11). Previous research has revealed that it can improve patient survival and quality of life, increase patient and family knowledge, reduce complications, and decrease unplanned readmission rates (12, 13). However, our literature review indicated that there are very few studies on evaluation indicators of discharge planning (e.g., discharge readiness, self-management ability, and safe eating behaviors) for stroke patients with dysphagia. In addition, discharge planning is mostly performed during or after patients’ hospitalization, and there is a lack of research on evaluation indicators of discharge planning (e.g., discharge readiness, self-management ability, and safe feeding) for patients with swallowing disorders in stroke. Meantime, the community health service in China is not perfect, and there is a lack of proper communication and cooperation between hospitals and communities. This may lead to an aggravation of the disability degree of stroke patients, a recurrence of the disease or readmission. So, the discharge planning has become an essential service that healthcare facilities are required to provide to ensure patients receive continuous care post-discharge, as suggested by American Nurses’ Association (14). Therefore, this study developed a discharge planning for stroke patients with dysphagia based on evidence-based medicine. The impact of this protocol on unplanned readmission rate, aspiration, and improving discharge readiness, self-management ability, and safe feeding in patients with stroke dysphagia was evaluated in a prospective cohort study.

## 2 Methods

### 2.1 Study design and setting

A prospective cohort study was conducted in a tertiary hospital in Yichang, Central China, from May 2023 to January 2024. This study was approved by the Ethics Committee of the corresponding author’s hospital (IRB No. PJ-KY2023-15) and registered at the Chinese clinical trial registry (ChiCTR24000087983).

### 2.2 Participants

Participants were recruited from May to December 2023, with follow-up ending in January 2024. The first eligible patient was enrolled on May 16, 2023.

The inclusion criteria were as follows:

1. 18 years of age or older.
2. Meeting the diagnostic criteria for stroke (15) and confirmed by head CT or MRI.
3. Standardized Swallowing Assessment (SSA) score ranging from 19 to 46.
4. Clear consciousness and normal cognitive function.
5. Contactable by phone after discharge.
6. Able to use WeChat.
7. Not participating in other research projects.
8. Willing to participate in this study and sign the informed consent form.

The exclusion criteria were:

1. Patients with consciousness impairment, cognitive dysfunction, or psychiatric disorders, or those who will be transferred to another medical institution after discharge.
2. Patients who are unable to cooperate with discharge follow-up.

The withdrawal criteria were:

1. Lost to follow-up patients.
2. Patients deemed unsuitable for continued participation by the researchers.

### 2.3 Data collection and management

Two trained research assistants, after obtaining explained the purpose of the study, written informed consent, independently collected data. They were blinded to the patients’ group allocation. Baseline data (T0) were collected at the time of patient admission. Data on unplanned readmission, aspiration, discharge readiness, self-management ability, and safe feeding were collected on the day of discharge (T1), 7 days after discharge (T2), and 30 days after discharge (T3).

We conducted thorough quality assurance on the reported dataset using advanced statistical software to identify any missing values, outliers, or anomalies. Patient identifiers associated with questionable data points were promptly forwarded to our local data collection team, who carefully reviewed the original sources to confirm data accuracy and identify any potential errors or omissions. Importantly, the final comprehensive review revealed no significant outliers in the dataset.

### 2.4 Sample size and grouping

The sample size was calculated by the two-sample mean calculation formula: N1 = N2 = 2 [(ta/2 + tβ/2)S/*δ*]^2^, using a two-sided alpha risk of 5% with a statistical power of 90%. This formula was obtained from a previous similar study (S = 4.8, δ = 35.35) (16), and the minimum sample size required was 36 per group. Thus, considering a 20% attrition rate, at least 90 participants were required (45 in each group).

The study was grouped according to wards, Neurology Ward 1 and Ward 2, to avoid contamination of the intervention program by contact communication between the exposed and non-exposed groups. Using natural grouping method, patients admitted to Ward 1 were assigned to the exposed group, and patients admitted to Ward 2 were assigned to the non-exposed group, based on the inclusion and exclusion criteria.

### 2.5 Intervention

Prior to initiating clinical practice, the research team developed a discharge planning through a multi-step process. First, the team conducted a systematic evaluation to summarize the best evidence on the clinical management of dysphagia in stroke patients and combined it with the best evidence on the key tasks of inpatient discharge planning (17) to develop a first draft of a discharge planning protocol for stroke patients with dysphagia. Subsequently, 10 experts from the departments of neurology, rehabilitation, nutrition, ear, nose and throat, and care management were invited to provide feedback on the initial draft. Based on the experts’ comments, the initial draft of the discharge planning was revised and improved, and the final version was developed. The program consisted of 4 phases (Supplementary material Table 1). The exposed group received the discharge planning constructed by the research team in addition to standard care. The non-exposed group received routine health education during the admission and hospitalization phases. On the day of discharge, patients of both groups will receive a discharge summary (including diagnostic and treatment records, post-discharge medication, dietary and exercise recommendations, etc.) and verbal discharge instructions provided by the nurse. Nurses will conduct telephone follow-up visits at 7 and 30 days after discharge, which will include inquiring about the patient’s physical condition, reminding him/her to follow up regularly, and providing him/her with the telephone number of the hospital follow-up center for easy access to consultation.

### 2.6 Fidelity of program implementation

Several strategies were adopted to the discharge planning fidelity. First, implementers were trained and asked to follow the program protocol strictly. Second, discharge guidance manual for stroke patients with dysphagia and structured telephone follow-up record sheet were developed to standardize the patient education contents and telephone follow-up. Third, the research team met weekly to provide feedback on the implementation of the program and to resolve problems encountered during implementation.

### 2.7 Instrument with validity and reliability

The outcome variables of the study included unplanned readmission rate, incidence of aspiration, discharge readiness, self-management ability, and safe feeding, which were assessed by relevant scales.

#### 2.7.1 Unplanned readmission and aspiration

The unplanned readmission rate refers to an unexpected hospital readmission due to the same or related illness within a specified time after discharge, typically calculated as the unplanned readmission rate within 30 days of discharge (18). The aspiration refers to the ratio of the number of patients who experience aspiration to the total number of patients during a given time (19) and the data were collected via a self-designed record table.

#### 2.7.2 Discharge readiness

Discharge readiness was evaluated by the Readiness for Hospital Discharge Scale for stroke patients developed by Liu Xingchen (20). The scale consists of 29 items across 4 dimensions: physiological status, psychological status, discharge knowledge, and social support. Each item was scored using a 5-point Likert scale: completely agree, basically agree, agree, somewhat disagree, and completely disagree, with scores of 5, 4, 3, 2, and 1, respectively. The total score (range = 0–220) is calculated by summing the 29 items, with higher scores indicating better discharge readiness. Cronbach’s *α* coefficient for this scale was 0.905.

#### 2.7.3 Self-management ability

Self-management ability was evaluated by the Stroke Self-Management Behavior Scale developed by Yanqiao Wang (21), which consists of 51 items. Each item is rated on a five-point scale: A = 1 point, B = 2 points, C = 3 points, D = 4 points, and E = 5 points. The total score (range = 51–255) is obtained by summing the scores of the 51 items, with higher scores indicating better self-management behavior. The scale includes 7 dimensions: disease management, medication safety management, dietary management, daily life management, emotional management, social function and interpersonal management, and rehabilitation exercise management, and each dimension reflects the self-management behaviors of stroke patients in a specific domain. Cronbach’s *α* coefficient for this scale was 0.835.

#### 2.7.4 Safe feeding

Safe feeding was evaluated by the Stroke Dysphagia Patient Safe Feeding Assessment Scale developed by Xiaoyu Feng (22), which includes 6 dimensions: personal preparation, meal preparation, eating quantity and speed, food selection, post-meal handling, and post-meal observation. The scale consists of 24 items, each rated on a five-point Likert scale: “never,” “rarely,” “sometimes,” “often,” and “always,” scored from 1 to 5, respectively. All items were scored positively, with higher total scores indicating more safe feeding. Cronbach’s *α* coefficient for this scale was 0.931.

#### 2.7.5 Demographic and clinical data

Baseline demographic or clinical data, such as the participants’ gender, age, educational level, Primary caregiver, comorbidities, presence of indwelling devices, readmission risk, swallowing function, lesion location distribution, National Institutes of Health Stroke Scale (NIHSS), swallowing rehabilitation therapy and average length of hospital stays were collected.

### 2.8 Statistical analysis

All data were collated and quantified after validation by two people, then entered and stored in Microsoft Excel 2010. After confirming the accuracy of the data, statistical analysis was performed using R software (https://www.r-project.org/). For normally distributed data, statistical descriptions were given as means and standard deviations, while non-normally distributed data were described using medians and interquartile ranges. Differences between groups were compared using independent sample t-tests or Wilcoxon rank-sum tests. Categorical data were described using frequencies and percentages, with comparisons made using chi-square tests or Fisher’s exact tests. Repeated-measures information was analyzed using two-factor repeated-measures ANOVA. All statistical tests were two-sided, and the significance level was set at *α* = 0.05. Linear mixed-effects models (LMMs) were used to account for clustering effects in the longitudinal data, with a patient-level random intercept to control for within-individual correlations across four repeated measurements. To further assess robustness, generalized estimating equations (GEE) were performed as an independent analysis. In addition, four hierarchical mixed-effects models were constructed, sequentially adjusting for potential confounders.

### 2.9 Ethical considerations

This study was approved by the Ethics Committee of the corresponding author’s hospital (IRB No. PJ-KY2023-15) and registered at the Chinese clinical trial registry (ChiCTR24000087983). Participants were recruited from May to December 2023, with follow-up ending in January 2024. The first eligible patient was enrolled on May 16, 2023. All methods were performed in accordance with the Declaration of Helsinki. Ethical approval was received from the Institutional Review Board committee at the Yichang Central People’s Hospital. All participants received information about the study, including information that it was voluntary to participate and that they could withdraw their participation at any time with no further explanations. All participants signed the informed consent forms.

## 3 Results

### 3.1 Characteristics of the participants

**Figure 1:**
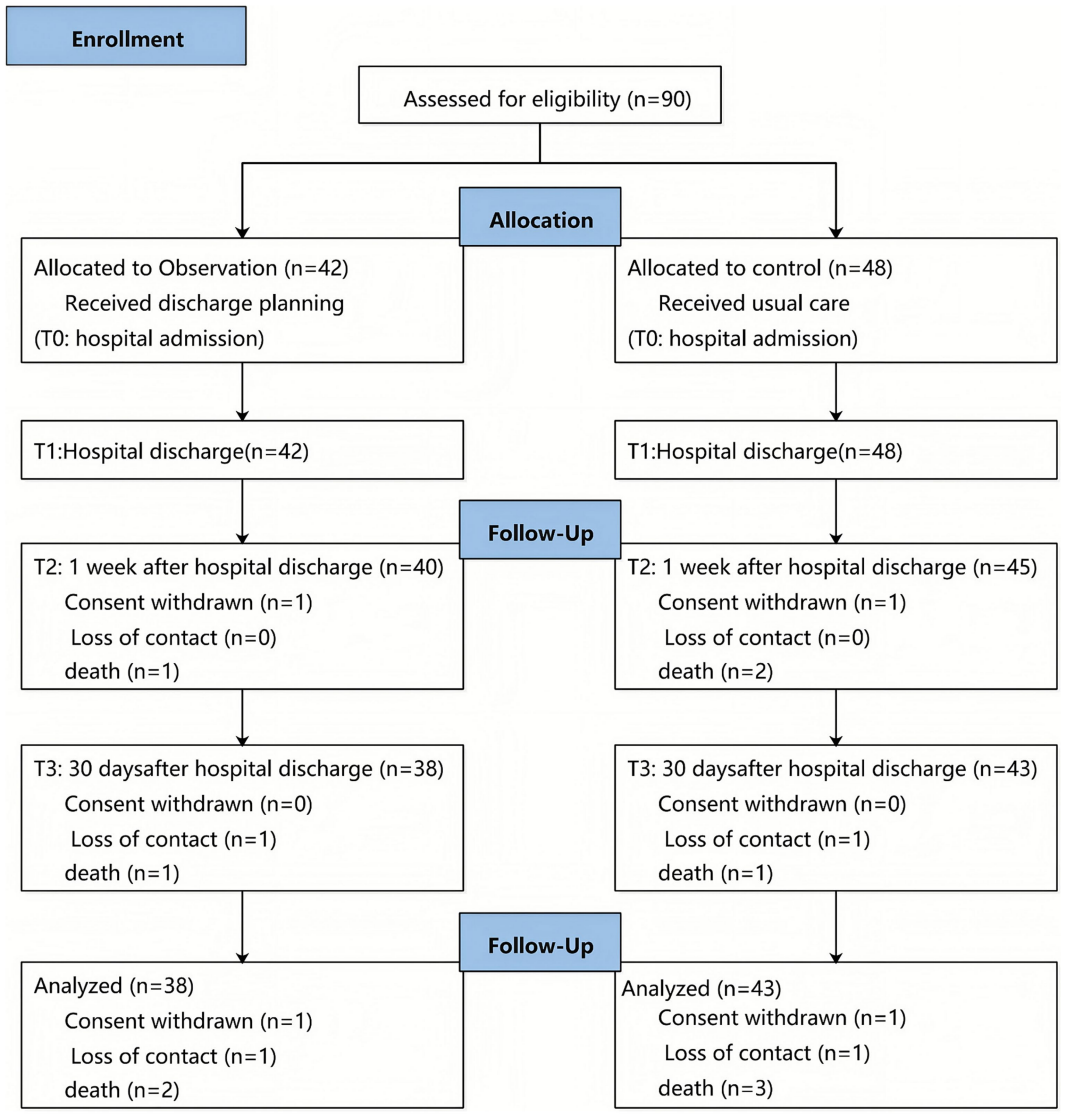
CONSORT flow diagram.

A total of 90 eligible participants were consecutively enrolled, of which 42 were in the exposed group and 48 were in the non-exposed group. All participants completed the data collection at hospital admission (T0) and at hospital discharge (T1). There were 9 participants dropped out of the study in total. Among them, 2 patients in the exposed group (4.7%) and 3 patients in the non-exposed group (6.3%) were lost to follow up at 7 days post-discharge (T2), for the loss rate of 5.6%. The drop-out rate at 30 days after discharge (T3) was 9.5% (4/42) in the exposed group and 10.4% (5/48) in the non-exposed group, and the total drop-out rate was 10%. Five patients in the non-exposed group dropped out for reasons including death due to illness (3 cases), inability to be contacted by phone (1 case) and transfer to a higher-level hospital (1 case). Four patients in the exposed group withdrew for reasons including death due to illness (2 cases), inability to be contacted by phone (1 case) and voluntary withdrawal from the study (1 case). A Consolidated Standards of Reporting Trials flowchart is presented in Figure 1. The characteristics of the participants in the two groups are shown in Supplementary material Table 2. There was no significant difference of characteristics between the exposed group and non-exposed group, especially in average length of hospital stays, lesion location distribution, swallowing rehabilitation therapy and functional severity of the stroke which were related to the stroke.

### 3.2 Effect of the discharge planning on the rate of unplanned readmission and aspiration

**Figure 2:**
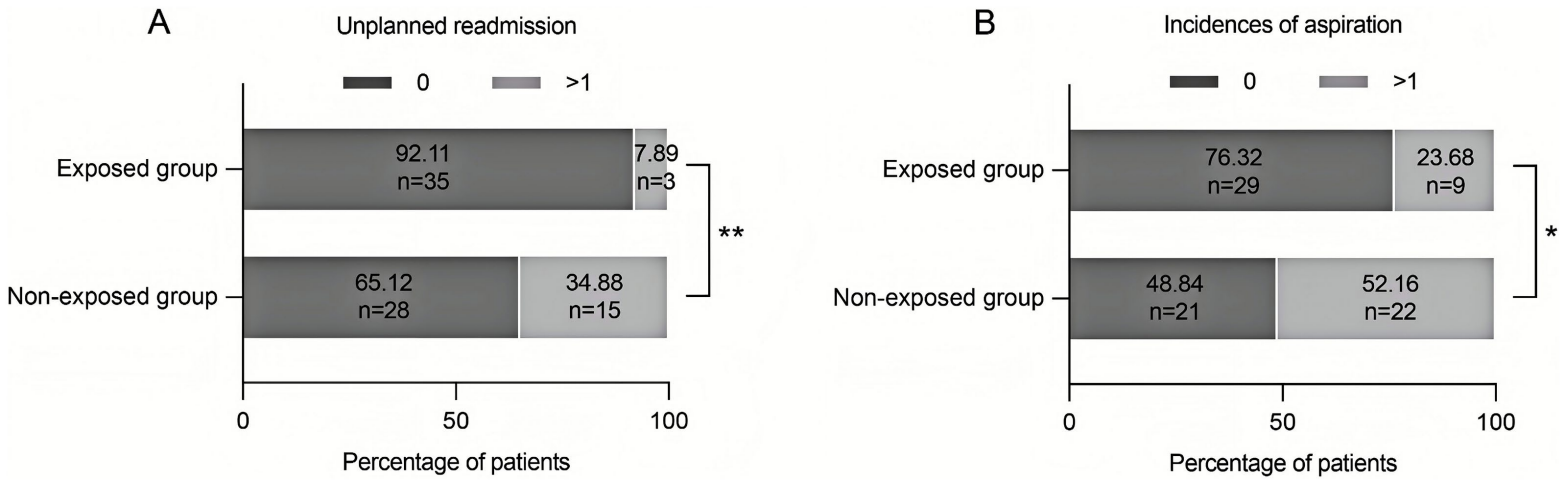
Improvement in prognosis of stroke patients with dysphagia by discharge planning. **(A)** The rate of unplanned readmission was significantly lower in the exposed group (7.89%) than in the non-exposed group (34.88%) (**p < 0.01). **(B)** The incidence of aspiration in the exposed group was significantly lower in the exposed group (23.69%) than in the non-exposed group (51.16%) (*p < 0.05).

The results of the Chi-square test showed that the rate of unplanned readmission and aspiration in the exposed group were significantly lower than those in non-exposed group (*p* < 0.05) (Figure 2).

### 3.3 Effect of the discharge planning on the readiness

**Figure 3:**
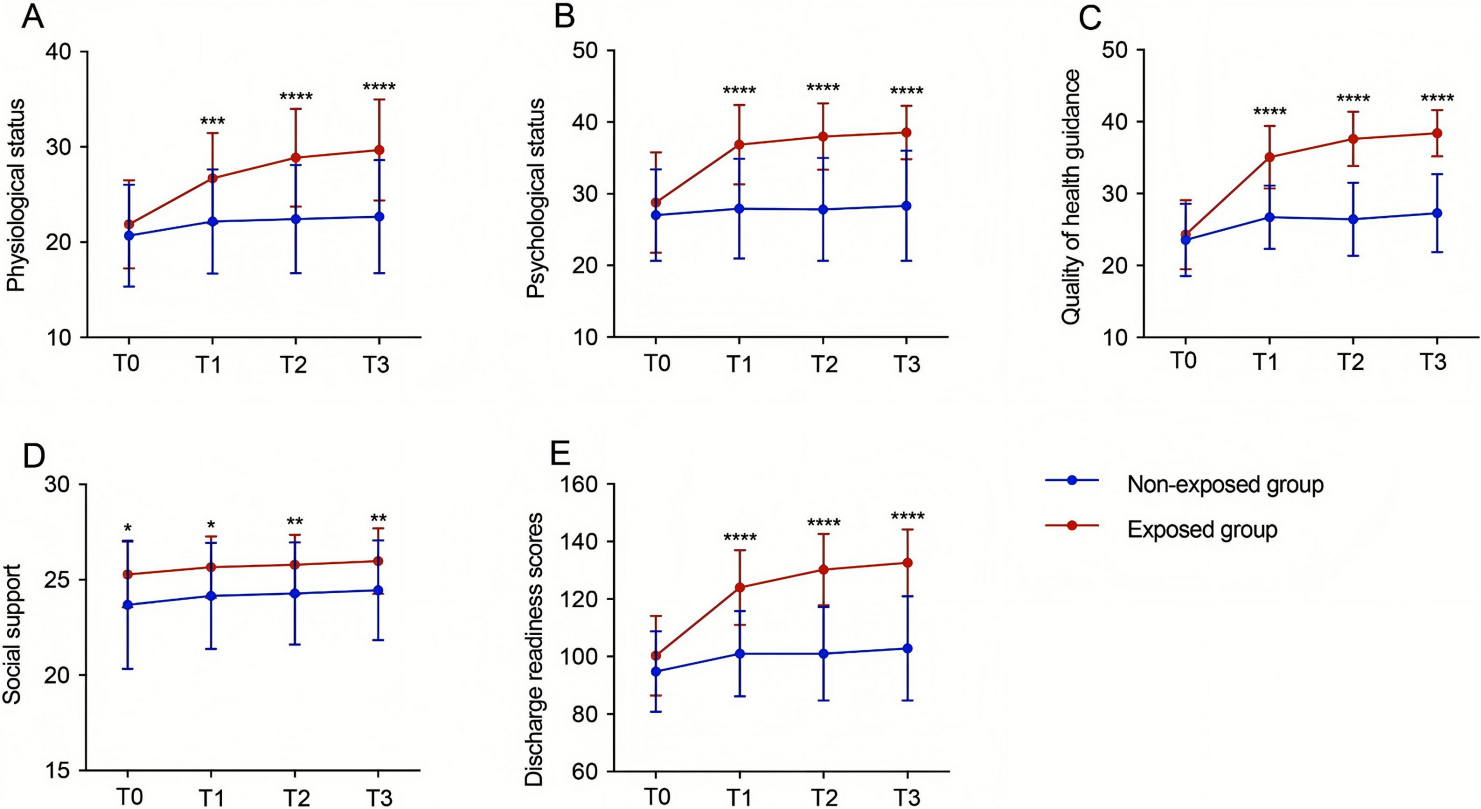
Improvement in discharge readiness of stroke patients with dysphagia by the discharge planning. Differences in physical status **(A)**, psychological status **(B)**, quality of health guidance **(C)** and social support **(D)**, and total discharge readiness score **(E)** among patients in the exposed group and patients in the non-exposed group at different time points(*p < 0.05, **p < 0.01, ***p < 0.001, ****p < 0.0001). T0: at admission, T1: on the day of discharge, T2: 7 days after discharge, T3: 30 days after discharge. A: F intergroup = 18.61, P intergroup < 0.00001, F time = 87.86, P time < 0.00001, F interaction = 31.17, P interaction < 0.00001; B: F intergroup = 37.13, P intergroup < 0.00001, F time = 52.44, P time < 0.00001, F interaction = 33.38, P interaction = 0.0030; C: F intergroup = 79.37, P intergroup < 0.00001, F time = 184.9, P time < 0.00001, F interaction = 67.53, P interaction < 0.00001; D: F intergroup = 9.581, P intergroup = 0.0027, and F time = 7.633, P time = 0.0033, F interaction = 0.037, P interaction = 0.9904; E: F intergroup = 53.31, P intergroup = 0.0003, F time = 171.4, P time < 0.00001, F interaction = 66.94, P interaction < 0.00001.

The results of the two-way repeated measures ANOVA showed that there was no significant difference between the two groups in the three dimensions (physical status, psychological status, and quality of health guidance) at baseline, but there was a significant difference in the social support dimension (*p* < 0.05). At T1, T2, and T3, the scores of the four dimensions (physical state, psychological state, quality of health guidance, social support) and the total score of discharge readiness were significantly higher in the exposed group than in the non-exposed group (*p* < 0.01) (Figure 3).

### 3.4 Effect of the discharge planning on the self-management ability

**Figure 4:**
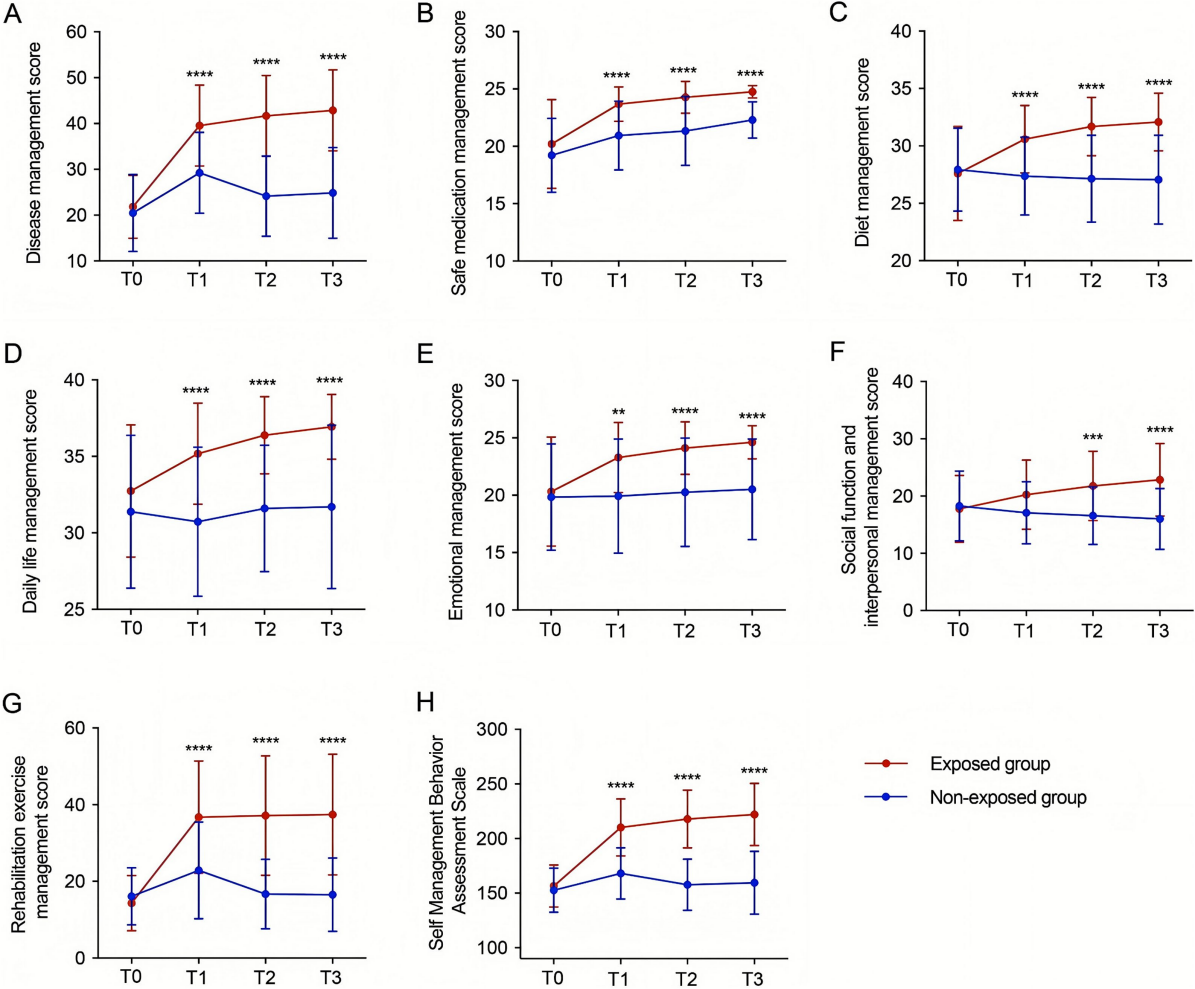
Improvement in self-management ability of stroke patients with dysphagia by discharge planning. Differences in the scores of patients in the exposed group and patients in the non-exposed group at different time points in the 7 dimensions of disease management **(A)**, safe medication management **(B)**, dietary management **(C)**, daily life management **(D)**, emotional management **(E)**, social functioning and interpersonal management **(F)**, and rehabilitation and exercise management **(G)**, as well as the total score **(H)** (**p < 0.01, ***p < 0.001, ****p < 0.0001). T0: at admission, T1: on the day of discharge, T2: 7 days after discharge, T3: 30 days after discharge. A: F intergroup = 56.41, P intergroup < 0.00001, F time = 90.52, P time < 0.00001, F interaction = 34.40, P interaction < 0.00001; B: F intergroup = 26.62, P intergroup < 0.00001, F time = 69.1, P time < 0.00001, F interaction = 4.785, P interaction = 0.0030; C: F intergroup = 21.05, P intergroup < 0.00001, F time = 16.76, P time < 0.00001, F interaction = 36.59, P interaction < 0.00001; D: F intergroup = 26.12, the P intergroup < 0.00001, F time = 12.31, P time < 0.00001, F interaction = 9.179, P interaction < 0.00001; E: F intergroup = 14.71, P intergroup = 0.0003, F time = 16.19, P time < 0.00001, F interaction = 9.296, P interaction < 0.00001; F: F intergroup = 9.315, P between groups < 0.00001, F time = 5.583, P time = 0.0043, F interaction = 35.75, P interaction < 0.00001; G: F between groups = 37.13, P between groups < 0.00001, F time = 59.09, P time < 0.00001, F interaction = 39.44, P interaction < 0.00001; H: F between groups = 80.67, P between groups < 0.00001, F time = 106.7, P time < 0.00001, F interaction = 64.80, P interaction < 0.00001.

The results of the two-way repeated measures ANOVA demonstrated that there was no significant difference between the two groups in the dimensions and the total score of self-management behaviors at T0. At T1, the scores of 6 management dimensions (disease, safe medication, diet, daily life, emotion, rehabilitation exercise) were significantly higher in the exposed group than those in the non-exposed group (*p* < 0.01). However, there were no significant differences between the two groups in social functioning and interpersonal management. At T2 and T3, the total score of the self-management competence scale and the scores of the 7 dimensions were significantly higher in the exposed group than those in the non-exposed group (*p* < 0.01) (Figure 4).

### 3.5 Effect of the discharge planning on safe feeding

**Figure 5:**
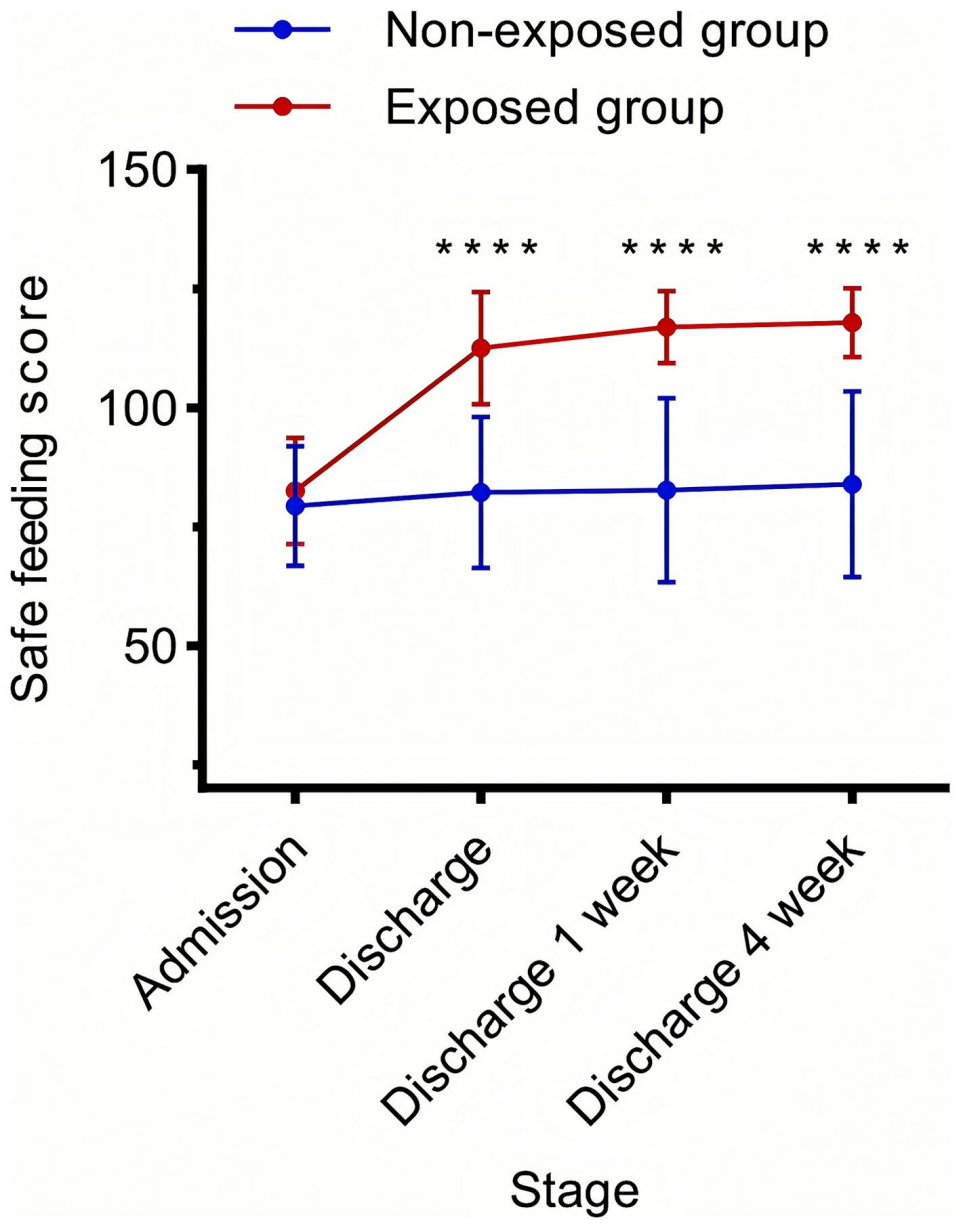
Improvement of safe feeding in stroke patients with dysphagia by the discharge planning. Differences in safe feeding scores of patients in the exposed group and patients in the non-exposed group at different time points (****p < 0.0001). T0: at admission, T1: on the day of discharge, T2: 7 days after discharge, and T3: 30 days after discharge. F Between Groups = 84.97, P Between Groups < 0.00001, F Time = 116.6, P Time < 0.00001, F Interaction = 74.69, P interaction < 0.00001.

The results of the two-way repeated measures ANOVA demonstrated that there was no significant difference between the two groups at T0. The scores of safe feedings were significantly higher in the exposed group than those in the non-exposed group at T1, T2, and T3 (*p* < 0.01) (Figure 5).

### 3.6 Clustering effects and sensitivity analysis

Linear mixed-effects models (LMMs) with patient-level random intercepts (ICC = 0.66) showed a significant group × time interaction (*β* = 7.85, 95% CI: 7.03–8.67; *p* < 0.001). Sensitivity analysis using GEE was consistent (*β* = 8.07, SE = 0.80; *p* < 0.001). Meantime, four hierarchical LMMs sequentially were adjusted for demographics, clinical factors, and lesion location. The group × time interaction remained significant across all models (*p* < 0.001), with Model 3 providing the best fit. In Model 3, the intervention group improved 7.85 points per time point compared with controls. NIHSS score (β = −0.62, *p* < 0.001) and indwelling catheters (*β* = −3.89, *p* < 0.001) were significant covariates; age and sex were not. Details are shown in Supplementary material S4.

### 3.7 Effect size and clinical significance

Collectively, multiple effect size indicators demonstrate that the evidence-based discharge planning program yields statistically significant and clinically meaningful improvements in discharge readiness among stroke patients with dysphagia. At 1 month, the Cohen’s d reached 1.919, indicating a “very large effect” far exceeding the magnitude typically observed in clinical interventions. The standardized interaction effect was 0.982, reflecting nearly one standard deviation of improvement at each time point. The intervention group improved 7.85 points more than the control group per time point, resulting in an accumulated gain of approximately 30 points across four measurements. The absolute between-group difference at 1 month was 30.09 points—equivalent to 20.1% of the total scale score—well above the commonly accepted threshold for clinical significance (10–15%). Additionally, the number needed to treat (NNT) was approximately 3–4, indicating that treating three to four patients results in one additional patient benefiting from the intervention, reflecting excellent cost-effectiveness. Together, these findings confirm that the intervention is not only statistically significant but also clinically impactful. Details are shown in Supplementary material S5.

## 4 Discussion

To our knowledge, this is the first prospective cohort study to explore the application effect of an evidence-based discharge planning in stroke patients with dysphagia. The target population was chosen because evidence shows that they are facing many health problems after stroke, especially during the period of transition from hospital to home. Our finding confirmed that evidence-based discharge planning is beneficial in reducing the unplanned readmission and aspiration, as well as improving patients’ discharge preparation, self-management and safe feeding.

Stroke patients with dysphagia may be readmitted to the hospital within a short period of time (23), and 40–90% of readmission are unplanned (24). Our finding is consistent with previous studies conducted by Kun et al. (25). Discharge planning is crucial for patients’ recovery, which can help patients better adapt to home rehabilitation and facilitate healthcare professionals to monitor patients’ condition changes. However, the nursing plan and medication management cannot be adjusted in time, and this may increase the risk of the patient’s readmission due to the inadequate preparation for discharge (26). The discharge planning for stroke patients with dysphagia provides dynamic assessment for swallowing function, nutritional status, readiness for discharge, self-management ability, and safe feeding through multidisciplinary collaboration. In addition, patients and their family members were instructed in rehabilitation exercises, medication compliance, diet, and daily living and daily life management through WeChat groups and telephone-based follow-up, which helps patients smoothly transit from inpatient to home state and reduce the unplanned hospital readmission.

Aspiration is a common and fatal complication for patients with stroke dysphagia, and more than 50% of patients develop dysphagia within 72 h after stroke (22), of which aspiration occurs in about 50% (27). Some studies have shown (28–31) that swallowing rehabilitation, therapy and dietary management for stroke patients can improve swallowing function, increase the proportion of safe feeding and reduce the incidence of aspiration. Our finding showed that the aspiration rate in the exposed group (23.69%) was significantly lower than that in the non-exposed group (51.16%), indicating evidence-based discharge planning can improve the aspiration, which is consistent with the results of the above study. The effectiveness of our program might be attributed to some reasons as following: (1) Multidisciplinary team including neurologists, rehabilitation therapists, ENT specialists, and Brain-Heart health manager formulated personalized rehabilitation plans for the patients as early as possible according to the result of functional assessment. (2) After assessing the self-management ability and safe feeding, our team work out the nursing care plans and health guidance for patients; (3) The Brain-Heart health manager conducts health education for the patients and establishes a management manual for patients with swallowing disorders in stroke, which covers health education, rehabilitation guidance, prevention of complications, and rehabilitation treatment and medication use during the period of hospitalization. It is convenient for patients to check at any time and provides guidance for patients’ subsequent rehabilitation.

Discharge readiness is an important indicator for evaluating the quality of discharge planning. It can show us that whether the patient is able to safely transition home and has been equipped to rehabilitate and reintegrate into society after discharge (32). The results of this study showed that discharge planning can improve the readiness. This suggests that the discharge planning can not only help patients understand stroke-related knowledge but also improve their self-management ability after discharge (33, 34). In this study, patient’s physical condition, psychological state and quality of healthcare were improved at T1, T2, and T3 in exposed group. These finding suggests that discharge planning can improve the physiological and psychological status of stroke patients with dysphagia. The effectiveness of our program might be attributed to some reasons as following: (1) patients were comprehensively and scientifically assessed from the beginning of their admission to the hospital, and appropriate assessment tools were used. (2) Nursing care was tailored to the needs of the patients and their families, with a focus on individualized health education and systematic rehabilitation guidance. This helped patients and their primary caregivers make a smooth transition, while providing relevant referrals to improve discharge readiness. There is no way that patients’ social relationships and the objective medical environment can be improved by short-term interventions, which is consistent with the results of this study.

Self-management refers to the gradual development of an individual’s ability to manage disease symptoms, treatment, physiological, psychological, social changes, and lifestyle changes while coping with the progression of his or her own chronic disease (35). The results of a randomized controlled trial conducted by the scholars of Markle et al. (36) on 90 patients with stroke showed that post-discharge management of the patients could improve their self-management ability. Zhan et al. (37) could improve the self-health management ability of stroke patients and effectively improve the quality of life by continuing nursing intervention for 126 stroke patients. The results of this study suggest that the overall situation of self-management ability of patients in the exposed group is higher than that of the non-exposed group, especially in disease management and daily life management. Through the intervention of discharge planning, nursing staff can promote patient’s compliance behavior and ability to manage their own diseases when they provide comprehensive assessment and disease-related health education. However, patient’s social functioning and interpersonal management were not improved in duration of hospital stay. This may be related to patient’s poor adaptation and higher average age. Therefore, healthcare professionals should pay special attention to the social functioning and interpersonal management of elderly patients, encouraging them to engage in social interactions and care activities to enhance the efficiency and quality of care.

Dysphagia after stroke may lead to complications such as aspiration pneumonia and malnutrition (38). However, the key strategy to control these complications lies in improving patients’ safe feeding (39). Many factors can influence safe feeding, including health beliefs, self-efficacy and home care (40). Li et al. (41) demonstrated a significant reduction in the incidence of aspiration pneumonia by providing safe eating techniques and swallowing rehabilitation to 108 patients with dysphagia after acute stroke. Our results showed that discharge planning can improve patients’ safe feeding, which is consistent with the above findings. The main reasons are as follows: (1) Our discharge planning was based on the latest clinical evidence and was specifically designed for stroke-related swallowing disorders. (2) The rehabilitation therapist develops a personalized rehabilitation treatment plan for the patient based on the best evidence. (3) Brain-Heart Health Managers provided dietary management and eating instructions based on the best practices for dysphagia care and dynamically adapted the plan according to the follow-up. Therefore, the nursing-led multidisciplinary discharge planning can effectively improve safe feeding and reduce the occurrence of aspiration.

The principal findings of this study demonstrate strong statistical significance, substantial clinical relevance, and high robustness across multiple validation strategies. First, the intervention effect was pronounced, with a significant group × time interaction (*β* = 7.85, 95% CI: 7.03–8.67; *p* < 0.001), indicating that the intervention group improved an additional 7.85 points per time point compared with the control group, with benefits accumulating over time. Second, the clinical impact was considerable: at 1 month, the intervention group outperformed the control group by 30.09 points, representing 20.1% of the total scale score, accompanied by a very large effect size (Cohen’s *d* = 1.919) and an excellent number needed to treat (NNT = 3–4). Furthermore, the findings remained consistent across four hierarchical mixed-effects models and were corroborated by GEE analyses, demonstrating robustness to covariate adjustments and lesion location differences. Finally, the study employed rigorous methodological procedures, including appropriate handling of longitudinal clustering effects (ICC = 0.66), systematic adjustment for potential confounders, and comprehensive model diagnostics to ensure the validity of statistical inference. Together, these strengths reinforce the credibility of the study conclusions.

## 5 Limitations

There are several limitations to this study. First, participants of this study were recruited from one general tertiary hospital in one city in central China, which might limit the generalization of the results. In the future, our team will conduct a multi-center trial covering different regions and different levels of hospitals to validate the findings of this study. Second, 30-day follow-up period was relatively short. Long-term effectiveness of discharge planning was not confirmed. Third, due to the limited clinical resources, a prospective cohort study design was used in this study. A randomized controlled trial should be used in the future. Fourth, this study has not yet considered the impact of the implementation of the protocol on the workload of nursing staff, which can be further refined in the future.

In our study, the swallowing function assessment method has some methodological limitations. SSA and self-designed form are semi-quantitative tools. Their results cannot be directly compared with imaging gold standards such as the Video Fluoroscopic Swallowing Study (VFSS) or Fiberoptic Endoscopic Evaluation of Swallowing (FEES). Consequently, these tools cannot objectively identify key events such as “aspiration” or “pharyngeal residue,” which limits the objectivity of the assessment. Due to the lack of objective standard validation, the study may overestimate the diagnostic efficacy of SSA and the self-designed form. Although our study design offers convenience and feasibility, VFSS or FEES is obviously better in terms of scientific rigor. Future studies should compare these tools with gold standards to validate their cut-off values and clinical utility.

## 6 Conclusion

This study reveals that the evidence-based discharge planning is effective in improving in discharge readiness, self-management ability, and safe feeding, and reducing the incidence of unplanned readmission and aspiration in stroke patients with dysphagia. The multidisciplinary team, which includes specialists in area of swallowing function, nutrition, and rehabilitation therapy, performs dynamic interventions throughout the period from patients’ admission to follow-up. It is recommended that such a process should be integrated into the stroke patients’ care in clinical practice to ensure successful transitions from hospital to home.

## Data Availability

The datasets presented in this study can be found in online repositories. The names of the repository/repositories and accession number(s) can be found in the article/Supplementary material.
