# Supplementary material for: The effect of evidence-based discharge planning on the health outcomes of stroke patients with dysphagia: a prospective cohort study
Source: Front Neurol. 2026 Jan 7;16:1707847. doi: 10.3389/fneur.2025.1707847 (PMC12819585; doi:10.3389/fneur.2025.1707847)
Supplement: Supplementary file 1 [file Data_Sheet_1.zip › supplementary materials/S4 Effect Size and Clinical Significance.docx]

**S4 Effect Size and Clinical Significance**

Table 7. Effect sizes and clinical significance metrics

| Measure | Value | Interpretation |
| --- | --- | --- |
| Cohen's d at 1 month | 1.919 | Very large effect |
| Standardized interaction effect | 0.982 | Per-unit time advantage |
| Improvement rate difference | 7.85 points/timepoint | Additional improvement per time unit |
| Absolute difference at 1 month | 30.09 points | Total cumulative benefit |
| Relative improvement (% of scale) | 20.1% | Clinical significance indicator |
| Number Needed to Treat (NNT) | 3-4 | Patients needed to treat for one benefit |

A range of effect size metrics was summarized to evaluate the practical significance of the intervention effects.
