# Supplementary material for: The effect of evidence-based discharge planning on the health outcomes of stroke patients with dysphagia: a prospective cohort study
Source: Front Neurol. 2026 Jan 7;16:1707847. doi: 10.3389/fneur.2025.1707847 (PMC12819585; doi:10.3389/fneur.2025.1707847)
Supplement: Supplementary file 1 [file Data_Sheet_1.zip › supplementary materials/S3 Clustering Effects and Sensitivity Analysis.docx]

**S3. Clustering Effects and Sensitivity Analysis**

1.1 This study used linear mixed-effects models (LMMs) to account for clustering effects in the longitudinal data. Since each patient was measured repeatedly at four time points, there was correlation among observations. A patient-level random intercept was included in the model to control for within-individual correlations. The intraclass correlation coefficient (ICC) indicated that 66.3% of the variance was attributable to differences between patients, confirming the necessity and appropriateness of using a mixed-effects model. **Table 5** presents the detailed variance decomposition results. The between-patient variance (τ²) was 52.97, the within-patient variance (σ²) was 26.95, and the total variance was 79.92. The ICC was 0.6629, and the design effect was 2.9888, indicating that ignoring the clustering effect would underestimate the standard error by approximately threefold. Regarding ward-level clustering, since all patients were from the same standardized ward and received identical care procedures, between-ward differences were minimized. Therefore, patient-level random effects were sufficient to control for clustering in this study.

Table 3. Variance decomposition and intraclass correlation

| Component | Value | Interpretation |
| --- | --- | --- |
| Between-patient variance (τ²) | 125.65 | 66.3% of total variance |
| Within-patient variance (σ²) | 63.89 | 33.7% of total variance |
| Total variance | 189.55 | Sum of variance components |
| Intraclass Correlation (ICC) | 0.6629 | Proportion of variance between patients |
| Design Effect | 2.9888 | Adjustment factor for clustering |
| All patients were from the same standardized neurology unit, minimizing ward-level clustering | | |

1.2 To further assess the robustness of the results, generalized estimating equations (GEE) were performed as an independent analysis. The GEE method has less stringent assumptions regarding clustering and provides more robust estimates.The group × time interaction effects obtained from the two methods were highly consistent (LMM: β = 7.85, 95% CI: 7.03–8.67; GEE: β = 8.07, SE = 0.80), both reaching statistical significance (p < 0.001). This consistency further supports the robustness of the main findings, indicating that the results are not affected by model assumptions. The results from both LMM and GEE are shown in **Table 4**.

Table 4. Robustness check: LMM vs GEE results

| Parameter | Linear Mixed Model β (95% CI) | GEE β (SE) | Agreement |
| --- | --- | --- | --- |
| Group effect | 6.54 (0.57 to 12.51) | 3.45 (2.68) | Consistent |
| Time effect | 2.59 (1.51 to 3.67) | 2.87 (0.61) | Consistent |
| Group × Time interaction | 7.85 (6.28 to 9.42) | 8.07 (0.83) | Consistent |
| GEE provides robust estimates less sensitive to clustering assumptions | | | |

1.3 **Covariate Adjustment**
To ensure the robustness of the study results, four hierarchical mixed-effects models were constructed, sequentially adjusting for potential confounders. This hierarchical approach allows a clear demonstration of the stability of the intervention effect across different levels of adjustment and assesses the influence of each covariate. The comparisons of the four models are shown in **Table 5**.

**Model 1** was unadjusted, including only the group × time interaction (β = 7.86, 95% CI: 7.03–8.68).

**Model 2** adjusted for demographic factors (age and sex) on top of Model 1 (β = 7.85, 95% CI: 7.02–8.67).

**Model 3**, the primary analysis model, further adjusted for clinical factors (NIHSS score, comorbidities, and indwelling catheters) (β = 7.85, 95% CI: 7.03–8.67).

**Model 4**, for sensitivity analysis, additionally adjusted for lesion location (β = 7.71, 95% CI: 6.86–8.56).

Based on AIC and BIC, Model 3 showed the best fit and was selected as the primary analysis model. The key finding is that the group × time interaction remained highly consistent and significant across all four models (p < 0.001), demonstrating that the intervention effect is robust to covariate adjustment.

Table 5. Model comparison for longitudinal discharge readiness

| Model | Group×Time β (95% CI) | P-value | AIC | BIC |
| --- | --- | --- | --- | --- |
| Model 1: Unadjusted | 7.85 (6.24 to 9.46) | <0.001 | 2,430 | 2,460 |
| Model 2: + Demographics | 7.85 (6.24 to 9.46) | <0.001 | 2,428 | 2,466 |
| Model 3: + Clinical factors (Primary) | 7.85 (6.28 to 9.42) | <0.001 | 2,409 | 2,451 |
| Model 4: + Lesion location (Sensitivity) | 7.85 (6.28 to 9.42) | <0.001 | 2,389 | 2,449 |

1.4 The full results of the primary model (Model 3) are shown in Table 6. The group × time interaction effect was β = 7.85 (p < 0.001), indicating that the intervention group improved an additional 7.85 points per time point compared with the control group. Other significant covariates included NIHSS score (β = -0.62, p < 0.001), suggesting that higher stroke severity was associated with lower discharge readiness, and indwelling catheters (β = -3.89, p < 0.001), indicating that patients with indwelling catheters had markedly lower discharge readiness. Age and sex did not have a statistically significant effect on discharge readiness. The model included patient-level random intercepts to control for within-individual correlations (ICC = 0.66), ensuring the validity of statistical inferences.

Table 6. Primary model: Fully adjusted mixed-effects analysis

| Variable | β (95% CI) | P-value |
| --- | --- | --- |
| (Intercept) | 109.80 (91.52 to 128.08) | <0.001^Note:^ |
| group_numeric | 6.54 (0.57 to 12.51) | 0.034* |
| time_numeric | 2.59 (1.51 to 3.67) | <0.001 |
| age_centered | -0.18 (-0.40 to 0.04) | 0.115 |
| gender | -4.48 (-10.32 to 1.37) | 0.138 |
| nihss_centered | -0.94 (-1.55 to -0.32) | 0.004** |
| comorbidity | 1.33 (-5.95 to 8.60) | 0.721 |
| indwelling_tube | -6.02 (-11.84 to -0.21) | 0.046* |
| group_numeric:time_numeric | 7.85 (6.28 to 9.42) | <0.001 |
| Model uses patient-level random intercepts to account for within-patient correlation (ICC=0.66) | | |
| ^Note:^* p<0.05; ** p<0.01; *** p<0.001 | | |

1.5 Model Diagnostics and Quality Control. The four key diagnostic plots of the primary analysis model (Model 3) are shown in Figure 6, used to assess the validity of model assumptions. Based on these plots, Model 3 met the main assumptions of the linear mixed-effects model (linearity, homoscedasticity, normality, and independence), indicating that the statistical inferences are reliable.


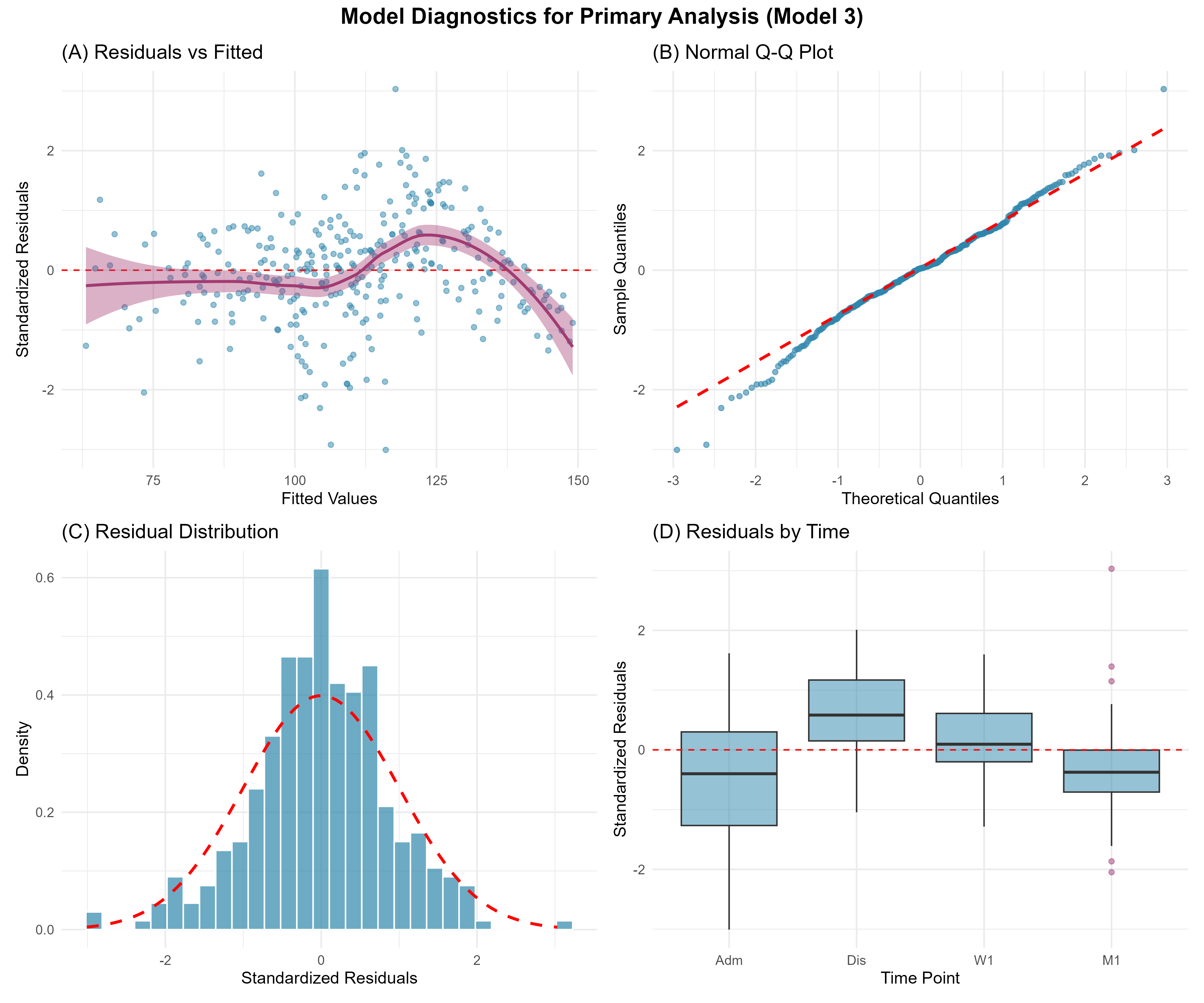


*Figure 6. Model Diagnostics for Primary Analysis (Model 3)*

(A) Residuals vs. Fitted Values Plot: Used to assess the homoscedasticity assumption. Ideally, residual points should be randomly scattered around the zero line without obvious fan-shaped or curved patterns. In this plot, the residuals are fairly evenly distributed, and the LOESS smoothed line (purple) is approximately horizontal, indicating that the homoscedasticity assumption holds. A few extreme points may represent special cases but do not affect the overall conclusion. (B) Q-Q Plot: Used to assess the normality of residuals. If the residuals follow a normal distribution, the points should lie close to the red dashed line (theoretical normal quantile line). Most points in this plot closely follow the theoretical line, with slight deviations at the tails, indicating good overall normality. The Shapiro-Wilk test (W = 0.9911, p = 0.0505) also supports the normality assumption (p > 0.05 threshold).(C) Histogram of Residuals: Provides a visual representation of the residual distribution. The blue bars represent the actual residuals, and the red dashed line represents the theoretical normal distribution curve. The close fit further confirms approximate normality of the residuals.(D) Residual Boxplots by Time Point: Used to check for systematic bias across different time points. The median residuals for all four time points are close to zero, and the box sizes are similar, indicating consistent model fit across time points with no time-dependent systematic error. A few outliers are highlighted in different colors and are within the normal range.
