# Supplementary material for: The effect of evidence-based discharge planning on the health outcomes of stroke patients with dysphagia: a prospective cohort study
Source: Front Neurol. 2026 Jan 7;16:1707847. doi: 10.3389/fneur.2025.1707847 (PMC12819585; doi:10.3389/fneur.2025.1707847)
Supplement: Supplementary file 1 [file Data_Sheet_1.zip › supplementary materials/S1 Table 1.Discharge planning program.doc]

**S1 Table 1. Discharge planning program.**

| **Time** | **Theme** | **Goal** | **Facilitator** | **Contents** |
| --- | --- | --- | --- | --- |
| Admission phase（Within the first 24 h after admission） | Swallowing Function Screening and Assessment | Identify patients with swallowing dysfunction | Primary nurse Brain-Heart Health Manager Speech Therapists | Assessment Content, Precautions, and Tools： 1.Screening of swallowing function, patient’s chief complaint, and detailed medical history, including relevant risk factors and physical examination. 2.Evaluation of the consistency and volume of fluids and foods swallowed, as well as the properties of medications taken. 3.Patients who test positive for swallowing disorders should undergo further evaluation by a speech therapist. 4.Clinical or instrumental assessment should be performed prior to treatment, with regular reassessment during treatment. 5.Patients with impaired consciousness should not undergo swallowing screening.6.Initial screening should be conducted using the SSA (Swallowing Screening Tool). |
|  | Nutritional screening and assessment | Identify patients at nutritional risk | Primary nurse | Assessment content, considerations, and tools: 1. For patients identified as having nutritional risk, a comprehensive nutritional assessment should be conducted by the multidisciplinary team. 2. Screening tool: Nutritional Risk Screening (NRS 2002). |
|  | Comprehensive assessment | Assess the patient's and caregiver's fundamental profile and individualized needs | Primary nurse Brain-Heart Health Manager  Speech therapist | Assessment content and considerations： 1.Physical functional status 2.Cognitive abilities 3.Mental/psychological state 4.Social and economic support 5.Expected professional care needs 6.Communication needs 7.Patient's treatment goals and preferences 8.Living environment 9.Readmission risk 10. Post-discharge services availability and feasibility 11.Caregiver's abilities and willingness 12.Medication history |
| Hospitalization phase | Formulate a plan | Develop an individualized discharge plan | Multidisciplinary team Patient family caregiver | Considerations：  1.Daily evaluation of the discharge plan 2.Develop intervention strategies and adjust the anticipated discharge date based on changes |
|  | Nutritional intervention | Improve the patient's nutritional status | Primary nurse Dietitian physician Speech Therapists | Nutritional interventions and considerations: 1.Prohibit the patient from eating or drinking until swallowing safety is assessed. 2.For patients with unsafe swallowing, modify the food texture and thicken liquids, encourage early oral intake, and regularly assess nutritional status. 3.For patients unable to swallow safely, use a nasogastric tube for short-term feeding (2-3 weeks) and recommend percutaneous endoscopic gastrostomy (PEG) for long-term feeding (≥4 weeks). 4.For swallowing dysfunction expected to last no more than 72 hours, consider short-term intravenous nutrition support. 5.Develop a phased nutritional management plan based on the patient's assessment and needs, and adjust it dynamically. 6.Use Fiberoptic Endoscopic Evaluation of Swallowing (FEES) to assess swallowing function, optimize food texture, and develop compensatory techniques and treatment plans. 7.In the recovery phase, intermittent tube feeding may be attempted to improve nutritional support. Select appropriate food textures, regularly monitor the effects of food adjustments, and ensure fluid balance and adequate nutritional intake. |
|  | Treatment and rehabilitation | Improve the patient's swallowing function | Speech Therapists physician Primary nurse Brain-Heart Health Manager | Rehabilitation therapy: 1.Treatment of swallowing disorders includes dietary adjustments, compensatory measures, rehabilitation training, nutritional support, improvements in feeding techniques, as well as nursing and pharmacological treatments. 2.Restorative swallowing exercises combined with compensatory techniques may be supplemented by adjunctive treatments such as neuromuscular electrical stimulation, repetitive transcranial magnetic stimulation (rTMS), and acupuncture. 3.Based on swallowing disorder assessments, develop an individualized treatment plan, flexibly incorporating rTMS or transcranial direct current stimulation (tDCS) in addition to standard treatments. 4.Improve swallowing function through swallowing muscle strength training and personalized techniques (e.g., Shaker exercise, Masako maneuver, Mendelsohn maneuver). 5.Design an electrical stimulation regimen with a frequency of 60-80 Hz, pulse duration of 700 microseconds, 20-30 minutes per session, 5 sessions per week, with electrodes preferably placed on the anterior neck. |
|  | Complication prevention | Reduce complications associated with dysphagia | physician Brain-Heart Health Manager  Primary nurse | Complication prevention: 1. Personalized nursing care: Develop a nursing plan to prevent aspiration. 2. Use texture-modified diets or thickened liquids to reduce the risk of aspiration. 3. Implement oral care interventions to prevent aspiration. 4. Perform oral care twice daily, and for patients with severe swallowing difficulties or those unable to eat orally, use 0.12% chlorhexidine to clean the oral cavity. |
|  | Health education | Help patients acquire nutritional knowledge and feeding skills | Multidisciplinary team Patient family caregiver | Health education: 1. Teach patients and their families to prevent complications such as aspiration and malnutrition. 2. Nutritional education and dietary guidance: Instruct on food selection, preparation, and feeding techniques, including mealtime environment, posture, utensils, and control of food and fluid intake. |
| Predischarge phase（3 days before discharge） | Discharge preparation | Evaluate and improve the implementation of the discharge planning | physician Brain-Heart Health Manager Primary nurse | Discharge preparation: 1. Assess the patient's swallowing rehabilitation, compliance, nutritional status, and psychological condition within 48-72 hours of discharge. Adjust the discharge plan, create a list of recommendations, and transfer to the receiving institution. Dynamically adjust the discharge plan and provide recommendations for rehabilitation, medication use, nutritional guidance, and follow-up appointments to the receiving institution. 2. Remind patients and their families of important considerations, confirming their residence, discharge time, transportation arrangements, and subsequent care needs. 3. Provide a written discharge summary and verify whether the discharge planning has been fully completed. |
| Post-discharge phase（within 30 days after discharge） | Follow-up and evaluation | Assess the patient's and caregiver's transition phase status | Brain-Heart Health Manager Primary nurse physician | Follow-up at 1 week and 1 month post-discharge: 1 .Incidence of readmission and aspiration. 2.Patient's self-management abilities. 3.Patient's feeding and nutritional status. 4.Patient's and family's coping abilities regarding the illness. |
