# Supplementary material for: The effect of evidence-based discharge planning on the health outcomes of stroke patients with dysphagia: a prospective cohort study
Source: Front Neurol. 2026 Jan 7;16:1707847. doi: 10.3389/fneur.2025.1707847 (PMC12819585; doi:10.3389/fneur.2025.1707847)
Supplement: Supplementary file 1 [file Data_Sheet_1.zip › supplementary materials/S2 Table 2.Characteristics of the participants.doc]

**S2 Table 2. Characteristics of the participants.**

|  | | Non-exposed group (n=43) | Exposed group  (n=38) | *P* |
| --- | --- | --- | --- | --- |
| Gender (N, %) | Male | 29 (67.44) | 25 (65.79) | 1.000^a^ |
|  | Female | 14 (32.56) | 13 (34.21) |  |
| Age (N, %) | <70 | 16 (37.21) | 15 (39.47) | 1.000^a^ |
|  | >70 | 27 (62.79) | 23 (60.53) |  |
| Educational level (N, %) | Primary school and below | 13 (30.23) | 16 (42.11) | 0.667^a^ |
|  | Junior high school | 16 (37.21) | 11 (28.95) |  |
|  | Senior high school | 10 (23.26) | 9 (23.68) |  |
|  | College or university and above | 4 (9.30) | 2 (5.26) |  |
| Primary caregiver (N, %) | Married couple | 18 (41.86) | 26 (68.42) | 0.110^a^ |
|  | Children | 14 (32.56) | 6 (15.79) |  |
|  | Other caregivers | 11 (25.58) | 6 (15.79) |  |
| Comorbidities (N, %) | Yes | 34 (79.07) | 34 (89.47) | 0.332^c^ |
|  | None | 9 (20.93) | 4 (10.53) |  |
| Indwelling tube (N, %) | Yes | 21 (48.84) | 10 (26.32) | 0.064^a^ |
|  | None | 22 (51.16) | 28 (73.68) |  |
| Readmission risk (N, %) | Yes | 43 (100) | 38 (100) | N/A^c^ |
|  | None | 0 (0.00) | 0 (0) |  |
| Swallowing function  (Mean ± SD) |  | 23.26 ± 2.88 | 22.37 ± 2.24 | 0.123^b^ |
| Average length of hospital stays | <10 | 9 (20.93) | 8 (21.05) | 1.000^a^ |
|  | ≥10 | 34 (79.07) | 30 (78.95) |  |
| Lesion location distribution(N, %) | Basal ganglia | 20 (46.5%) | 27 (71.1%) | 0.161^a^ |
|  | Cerebellum | 2 (4.7%) | 2 (5.3%) |  |
|  | Frontal lobe | 12 (27.9%) | 5 (13.2%) |  |
|  | Multiple/Other | 8 (18.6%) | 3 (7.9%) |  |
|  | Pons | 0 (0.0%) | 1 (2.6%) |  |
|  | Temporal lobe | 1 (2.3%) | 0 (0.0%) |  |
| NIHSS, median(IQR) |  | 6 (10) | 3 (5) | 0.278^d^ |
| Swallowing rehabilitation therapy  (N, %) | Yes | 22(51.16) | 20(52.63) | 1.000^a^ |
|  | None | 21(48.84) | 18(47.37) |  |

^a^ Chi-squared test. ^b^ Independent-sample t-test. ^c^ Fisher exact test. ᵈ Mann-Whitney U test. N/A: identical distribution.
